# The performance of stroke survivors in turning-while-walking while carrying out a concurrent cognitive task compared with controls

**DOI:** 10.1371/journal.pone.0189800

**Published:** 2017-12-22

**Authors:** Wing-nga Chan, William Wai-nam Tsang

**Affiliations:** Department of Rehabilitation Sciences, Hong Kong Polytechnic University, Hong Kong SAR, China; Taipei Veterans General Hospital, TAIWAN

## Abstract

**Background:**

Turning-while-walking is one of the commonest causes of falls in stroke survivors. It involves cognitive processing and may be challenging when performed concurrently with a cognitive task. Previous studies of dual-tasking involving turning-while-walking in stroke survivors show that the performance of physical tasks is compromised. However, the design of those studies did not address the response of stroke survivors under dual-tasking condition without specifying the task-preference and its effect on the performance of the cognitive task.

**Objective:**

First, to compare the performance of single-tasking and dual-tasking in stroke survivors.

Second, to compare the performance of stroke survivors with non-stroke controls.

**Methods:**

Fifty-nine stroke survivors and 45 controls were assessed with an auditory Stroop test, a turning-while-walking test, and a combination of the two single tasks. The outcome of the cognitive task was measured by the reaction time and accuracy of the task. The physical task was evaluated by measuring the turning duration, number of steps to turn, and time to complete the turning-while-walking test.

**Results:**

Stroke survivors showed a significantly reduced accuracy in the auditory Stroop test when dual-tasking, but there was no change in the reaction time. Their performance in the turning-while-walking task was similar under both single-tasking and dual-tasking condition. Additionally, stroke survivors demonstrated a significantly longer reaction time and lower accuracy than the controls both when single-tasking and dual-tasking. They took longer to turn, with more steps, and needed more time to complete the turning-while-walking task in both tasking conditions.

**Conclusions:**

The results show that stroke survivors with high mobility function performed the auditory Stroop test less accurately while preserving simultaneous turning-while-walking performance. They also demonstrated poorer performance in both single-tasking and dual-tasking as compared with controls.

## Introduction

Turning-while-walking is a common daily activity [1]. It has been suggested that turning is not automatic but requires cognitive processing to provide feedback control [2]. Information from various sensory systems, including the visual system, vestibular system and somatosensory system, are integrated throughout the movement, and the body adjusts accordingly [3, 4]. In turning-while-walking, it has been suggested that failing to adjust the movement from the already coordinated straight line walking may result in loss of balance or even a fall [5]. Indeed, turning is among the activities most frequently reported to cause a fall in older people with stroke [3]. Falling during a turn is 7.9 times more likely to result in a hip fracture in stroke survivors than walking in a straight line [1]. Previous studies have shown that a stroke affects the performance of turning-while-walking. Stroke survivors took a longer time to turn and needed more steps than their age-matched counterparts [6, 7]. In addition, they turned more slowly with less displacement of the center of mass in the mediolateral direction [8]. The reorientation sequence of body segments was also disrupted [9].

Performing two tasks simultaneously, such as responding to an external distraction while walking, is common in everyday life. This dual-tasking ability has been shown to be related to both falls [10] and functional ability [11]. Based on the theory of competition for attentional resources, dual-tasking performance is suggested to be affected by several factors: 1) fewer attentional resources are available to perform two tasks concurrently, 2) inability to allocate resources between the two tasks correctly, 3) increased resource requirement to carry out the individual tasks, and 4) a combination of these factors [12]. If any of these factors are present, the performance of either or both tasks will be downgraded.

Many studies have been conducted to investigate dual-tasking ability in stroke survivors [10, 11, 13] but the number investigating turning-while-walking is limited. The available studies consistently showed that an additional cognitive task has a detrimental effect on the turning-while-walking performance [14–17]. However, there are questions that those studies could not answer. First, in some of the studies subjects were asked to prioritize the cognitive task over the physical task [14–16]. Therefore, it is not clear which task the subjects would naturally prioritize without this instruction. Further, the level of performance of the cognitive task under dual-tasking conditions was not given by the available studies. They either did not compare changes in cognitive task performance from single-tasking to dual-tasking [17] or did not consider the performance in the statistical analysis [14–16]. Therefore, it is not clear whether the subjects also compromised the cognitive task under dual-tasking conditions. Moreover, the cognitive task (serial subtraction) employed in these studies uses internal interference. The response of stroke survivors to a dual-tasking condition involving an external disturbance, which is common in daily life, is yet to be determined.

Therefore, this study was conducted to investigate the natural response of stroke survivors to a dual-tasking challenge involving turning-while-walking as the physical task. This study had two aims:

1. To compare single-tasking and dual-tasking performance in stroke survivors. We hypothesized that the performance of both tasks would be degraded when dual-tasking.
2. To compare the performance of stroke survivors with non-stroke controls. We postulated that stroke survivors would have a lower dual-tasking ability than the controls due to a decreased ability to conduct the individual tasks, as reflected by the single-tasking performance. We also hypothesized that the effect of dual-tasking would be greater in the stroke survivors than the controls, which was examined by comparing the dual-task cost between the two groups.

## Method

### Participants

Stroke survivors were recruited from local mutual self-help groups. The control subjects were recruited in the community. Eligible subjects were aged 50 or above, able to walk unaided for at least 15 meters indoor without physical assistance, and able to follow instructions. An additional criterion for subjects in the stroke survivor group was it having been at least six months since the onset of stroke. Subjects with any of the following conditions were excluded: a severe visual or hearing problem, a score of less than 18 in the Mini-Mental State Examination (MMSE) (Cantonese Version) [18], any neurological disease (other than stroke in the stroke survivor group) or any musculoskeletal trauma or medical operation within the previous six months. This study was approved by the Ethics Committee of the Hong Kong Polytechnic University. The aims and procedures were explained to all participants before they gave their informed consent.

### Procedures

Demographic data including gender, age, height, weight, and mobility function [the timed Up-and-Go test (TUGT) and the Breg Balance Scale (BBS)] were obtained before the assessment. For the stroke subjects, chronicity, side, number and type of stroke were collected. All of the subjects were assessed with a single cognitive task (auditory Stroop test), a single physical task (turning-while-walking test), and a combination of the two single tasks. Detailed procedures were explained and familiarization trials were given to all the subjects before the assessment. The sequence of testing the three conditions was randomized to minimize any learning effect.

#### Single auditory Stroop test

The auditory Stroop test was designed to assess the executive function, including working memory, selective attention, and ability to inhibit automatic responses [19, 20]. In this test, two pre-recorded words, “high” and “low,” were pronounced in two different pitches, high and low, to form four combinations. The subjects were instructed to ignore the meaning of the word and respond to its pitch as quickly and as accurately as possible. A two-button switch, with each button representing the high and low pitch respectively, was given to the subject to react to the auditory Stroop test. Stroke subjects held the switch with the less affected hand, while controls held it with the dominant hand (defined as the hand used for writing). Each combination of word and pitch was tested three times for each subject in a randomized sequence.

The reaction times and accuracy of the responses were recorded using custom-made software (LabVIEW version 8.6, National Instruments Corp., Austin, TX, USA). The reaction time was defined as the period from the start of the sound to the button press. The average reaction time of the 12 trials was calculated for data analysis. The accuracy was calculated by dividing the number of correct responses by the total number of trials.

#### Single turning-while-walking test

In the single turning-while-walking test, the subjects were asked to walk a 5-meter straight line, make a 180-degree turn, and then return to the starting position as quickly and safely as possible [21]. A force platform (Model OR6-5-1000, Advanced Mechanical Technologies Inc., Newton, MA, USA) was installed in the walking surface five meters from the starting position. As the force platform was used to trigger the auditory Stroop test in the dual-tasking condition, no sound was produced in this single-tasking test. The subjects should press any button on the switch used in the auditory Stroop test while turning to make the data comparable with those from the dual-tasking tests. A research assistant walked closely behind the subject during the assessment to ensure safety. The subjects were allowed to use any walking aids or wear their ankle-foot orthosis as preferred.

A gyroscopic sensor system for gait analysis (Mobility Lab iWalk, OPAL sensors, APDM Inc., Portland, OR, USA) was used to record the turning performance. Sensors on the trunk and the lumbar region detected the moment when turning started and ended. Any step with more than 50% of its phase within the turning period was counted as a step within the turn [22, 23]. The test was repeated three times. Averages of the turning duration, number of steps to turn, and duration of the turning-while-walking test were employed for data analysis.

The subjects with stroke were asked to turn towards the affected side, as falls are more common when turning to this side [24]. Control subjects were tested turning to both sides. However, there was no significant difference in performance between the two turning sides (*p* > 0.05). Thus, data from controls of all trials were combined for statistical analysis.

#### Dual-tasking test

In the dual-tasking test, subjects were required to perform the turning-while-walking test concurrently with the auditory Stroop test. The setting was the same as that of the single turning-while-walking test. The force platform installed on the walking surface triggered one of the four combinations of the sound of the auditory Stroop test when the subject stepped on it. Each of the four combinations was assessed once in a randomized sequence. The subjects were told that both tasks were equally important and no priority of the task was instructed. The parameters used in the single tasks were adopted in the dual-tasking condition. Any assistive devices used in the single-tasking test were used again.

#### Dual-task cost

The dual-task cost of each parameter of the cognitive task and the physical task was calculated to determine the change of performance between single-tasking and dual-tasking. As the single-tasking performance may vary between subjects, comparing the dual-task cost would take this baseline difference, if any, into consideration [25]. The dual-task cost was calculated as:

[(single-tasking–dual-tasking)/single-tasking]×100% (1)

For the accuracy measured by the auditory Stroop test, a positive value of the dual-task cost indicates a lower accuracy in the dual-tasking condition. For all other measures (reaction time of the auditory Stroop test, turning duration, number of steps to turn, and completion time of the turning-while-walking test), a negative dual-task cost value implies a decreased performance when dual-tasking.

#### Test-retest reliability

Test-retest reliabilities of the three tasking conditions were assessed. Thirteen stroke and nine control subjects were included. They were asked to take the assessments twice within two weeks. Subjects who had any change in medical, physical or cognitive condition or regular exercise habits within the test-retest period were excluded. The testing sequence in the retest session was identical to that of the first session.

### Statistical analysis

Commercial software, Statistical Package for the Social Sciences (SPSS)(version 23, IBM Corp., Armonk, NY, USA), was used for data analysis. Test-retest reliability was assessed with an intraclass correlation coefficient (ICC), model ICC (3,*k*), with ‘*k*’ as the number of trials in the tests being assessed. The difference in demographic characteristics between the stroke survivors and controls were assessed with independent t-tests. The sex distribution between the two groups was compared with a chi-square test. Two-way mixed multivariate analysis of variance (MANOVA) was employed to investigate the task effect (single-tasking and dual-tasking), the group effect (stroke survivors and controls), and the interaction effect (task x group). If results were significant, further data analysis was conducted using the paired t-test for significant task effect and the independent t-test for significant group effect. Bonferroni adjustments were applied for all follow-up analyses. To compare the dual-task cost between stroke survivors and the controls, independent t-test was employed. The statistical significance level was set at 0.05.

## Results

### Participants

Forty-five controls and 59 stroke survivors participated in this study. The demographic data are shown in Table 1. Gender, body height, MMSE score, TUGT, and BBS score were significantly different between the two groups.

**Table 1:** Demographics of the controls and stroke survivors.

		Control (n = 45)	Stroke (n = 59)	*p*-value
Gender (Male: Female)		9: 36	29: 30	0.004*
Age (years)		61.3 ± 4.8 (51–69)	62.4 ± 6.8 (51–77)	0.337
Height (cm)		158.1 ± 6.7 (145.0–171.0)	162.0 ± 8.5 (142.0–181.0)	0.016*
Weight (kg)		61.1 ± 10.1 (45.0–89.0)	61.8 ± 11.4 (41.0–100.0)	0.712
Years of education		10.1 ± 3.5 (0–16)	9.5 ± 4.1 (1–18)	0.525
MMSE		29.4 ± 1.1 (25–30)	27.9 ± 2.2 (21–30)	< 0.001*
TUGT (sec)		6.3 ± 1.0 (4.5–9.4)	18.0 ± 8.9 (5.5–44.3)	< 0.001*
BBS		55.9 ± 0.3 (55–56)	48.1 ± 6.8 (31–56)	<0.001*
Time since onset of stroke (years)		N/A	5.4 ± 4.8 (0.5–21.0)	N/A
Affected side	Right		25	
	Left		34	
Type of stroke	Ischemic		40	
	Haemorrhage		17	
	Both		2	
Number of strokes	1		49	
	2		8	
	3		2	

Values are in mean ± SD (range); MMSE: Mini-Mental Status Examination; TUGT: Timed Up-and-go Test; BBS: Berg Balance Scale

* denotes significant difference between stroke survivors and controls (*p* < 0.05)

### Test-retest reliability

Among the control subjects, the reliability of the three test conditions ranged from 0.72 to 0.91, indicating good to excellent reliability (Table 2). The intraclass correlation coefficient for the accuracy of the single-tasking auditory Stroop test could not be calculated as all subjects made no mistake, resulting in zero variance.

**Table 2:** Test-retest reliability in the controls.

		Time 1	Time 2	ICC (3,k)	95% CI	
					Lower	Upper
**Single-tasking**						
Auditory Stroop test	Reaction time (sec)	0.90 ± 0.20	0.93 ± 0.27	0.91	0.61	0.98
	Accuracy (%)	100.00 ± 0.00	100.00 ± 0.00	Zero variance		
Turning-while-walking	Turning duration (sec)	5.96 ± 0.70	5.98 ± 0.62	0.76	-0.08	0.95
	Number of steps to turn	8.95 ± 2.02	7.42 ± 1.25	0.89	0.51	0.98
	Completion time (sec)	2.48 ± 0.46	2.22 ± 0.26	0.83	0.24	0.96
**Dual-tasking**						
Auditory Stroop test	Reaction time (sec)	0.98 ± 0.30	0.92 ± 0.33	0.88	0.48	0.97
	Accuracy (%)	91.67 ± 8.84	97.22 ± 5.51	0.73	-0.36	0.95
Turning-while-walking	Turning duration (sec)	5.96 ± 0.58	6.05 ± 0.38	0.72	-0.23	0.94
	Number of steps to turn	8.55 ± 1.67	7.86 ± 2.32	0.90	0.55	0.98
	Completion time (sec)	2.36 ± 0.33	2.25 ± 0.25	0.78	0.01	0.95

Values are in mean ± SD

Among the stroke survivors, all of the parameters, except for the reaction time and accuracy of the auditory Stroop test under the dual-tasking condition, and the number of steps to turn in the single-tasking condition, indicated excellent reliability with ICC values over 0.90 (Table 2). The ICC (3,4) values for reaction time and accuracy in dual-tasking and the ICC (3,3) for number of steps to turn in single-tasking ranged from 0.62 to 0.85, indicating good reliability (Table 3).

**Table 3:** Test-retest reliability among the stroke survivors.

		Time 1	Time 2	ICC (3,k)	95% CI	
					Lower	Upper
**Single-tasking**						
Auditory Stroop test	Reaction time (sec)	1.14 ± 0.21	1.07 ± 0.16	0.95	0.77	0.99
	Accuracy (%)	91.03 ± 11.52	89.29 ± 12.47	0.94	0.72	0.99
Turning-while-walking	Turning duration (sec)	4.44 ± 1.41	4.24 ± 1.86	0.90	0.66	0.97
	Number of steps to turn	8.16 ± 1.51	8.00 ± 1.69	0.85	0.49	0.96
	Completion time (sec)	16.49 ± 6.80	15.50 ± 6.78	0.96	0.86	0.99
**Dual-tasking**						
Auditory Stroop test	Reaction time (sec)	1.12 ± 0.40	1.19 ± 0.31	0.62	-0.41	0.90
	Accuracy (%)	75.00 ± 28.87	85.42 ± 19.82	0.67	-0.24	0.91
Turning-while-walking	Turning duration (sec)	4.36 ± 1.74	4.11 ± 2.03	0.96	0.85	0.99
	Number of steps to turn	8.11 ± 2.13	7.73 ± 2.13	0.97	0.88	0.99
	Completion time (sec)	15.95 ± 6.66	15.48 ± 6.85	0.95	0.82	0.99

Values are in mean ± SD

### Comparing single-tasking and dual-tasking performance in stroke survivors

There was a significant difference between single-tasking and dual-tasking performance on the accuracy [*F*(1,99) = 12.175, *p* = 0.001], but not the reaction time [*F*(1,99) = 3.902, *p* = 0.051] of the auditory Stroop test. Follow-up analysis showed that stroke survivors made more errors under dual-tasking condition than in single-tasking (*p* = 0.001) (Table 4).

**Table 4:** Results of the single-tasking and dual-tasking performance among the controls and the stroke survivors.

		Controls (n = 45)		Stroke survivors (n = 59)		*F*-value (*p*-value)		
		Single-tasking	Dual-tasking	Single-tasking	Dual-tasking	Between- task difference	Between- group difference	Interaction effect
Auditory Stroop test	Reaction time (sec)	0.96 ± 0.22	1.02 ± 0.30	1.17 ± 0.33	1.22 ± 0.33	3.902 (0.051)	14.818 (< 0.001)^b^^,^ ^c^	0.021 (0.886)
	Accuracy (%)	98.15 ± 3.93	89.72 ± 11.70	91.02 ± 10.05	81.25 ± 21.98	12.175 (0.001)^a^	28.315 (< 0.001)^b^^,^ ^d^	0.214 (0.645)
Turning-while-walking	Turning duration (sec)	2.39 ± 0.44	2.41 ± 0.45	5.23 ± 2.43	5.35 ± 2.40	1.698 (0.196)	63.898 (< 0.001)^b^^,^ ^c^	0.773 (0.381)
	Number of steps to turn	6.10 ± 0.74	6.23 ± 0.64	9.06 ± 2.18	9.13 ± 2.35	1.270 (0.263)	63.898 (< 0.001)^b^^,^ ^c^	0.106 (0.746)
	Completion time (sec)	7.98 ± 1.68	8.01 ± 1.59	21.23 ± 12.97	21.25 ± 12.36	0.033 (0.857)	48.831 (< 0.001)^b^^,^ ^c^	0.000 (0.995)

Values are in mean ± SD

^a^ Significant difference between single-tasking and dual-tasking in controls and stroke survivors (*p* = 0.001)

^b^ Significant difference between controls and stroke survivors in single-tasking (*p* < 0.001)

^c^ Significant difference between controls and stroke survivors in dual-tasking (*p* < 0.001)

^d^ Significant difference between controls and stroke survivors in dual-tasking (*p* < 0.025)

By contrast with the cognitive task performance, no significant difference was found in the turning-while-walking performance between the single-tasking and dual-tasking conditions among the stroke survivors (*p* > 0.05).

### Comparing stroke survivors and controls

There were significant differences between the two groups in all the parameters of both the cognitive and physical tasks.

In the auditory Stroop test, stroke survivors showed a significantly longer reaction time (*p* = 0.003) and a lower accuracy (*p* = 0.015) than the controls when dual-tasking. Results with the same significance were also found in the single-tasking condition (both *p*-values = 0.001) (Table 4).

For the turning-while-walking performance under the dual-tasking condition, stroke survivors took both a longer turning time and more steps than the controls (both *p*-values < 0.001). Subjects with stroke also needed more time to complete the turning-while-walking test (*p* < 0.001). Similar differences between the two groups were also observed in the single-tasking condition (All *p*-values < 0.001) (Table 4).

Statistical comparison of dual-task cost revealed no significant difference in both the cognitive task and physical task between the stroke survivors and the controls (Table 5).

**Table 5:** Results of the dual-task cost.

		Controls (n = 45)	Stroke survivors (n = 59)	*p*-value
Auditory Stroop test	Reaction time (sec)	-7.5 ± 26.1	-8.1 ± 27.5	0.922
	Accuracy (%)	8.6 ± 11.7	10.7 ± 22.8	0.533
Turning-while-walking	Turning duration (sec)	-1.2 ± 7.5	-2.5 ± 11.9	0.546
	Number of steps to turn	-2.7 ± 8.9	-0.9 ± 11.6	0.397
	Completion time (sec)	-0.7 ± 5.1	-0.7 ± 8.6	0.985

Values are in mean ± SD

## Discussion

This study examined how stroke survivors performed in dual-tasking that involved an auditory Stroop test and a turning-while-walking test. The results show that when dual-tasking, stroke survivors made more errors in the cognitive task with similar turning-while-walking performance. Further, stroke survivors demonstrated a longer reaction time and a lower accuracy in the auditory Stroop test by comparison with controls. They also took longer to turn with more steps and needed more time to complete the turning-while-walking test. However, there was no significant difference in dual-task cost in both the cognitive task and physical task between the two groups.

In this study, stroke survivors responded to dual-tasking with reduced accuracy in the auditory Stroop test but with similar performance of the physical task. According to the theory of competition for attentional resources in dual-tasking [12, 26, 27], the results may imply that, at least in these conditions, stroke survivors may not have sufficient resources to perform both tasks concurrently. However, the hypothesis of interference between the cognitive and physical tasks when dual-tasking was only partly supported. This unexpected result can be explained by a posture-first strategy [27]. It is suggested that when dual-tasking, if the physical task is too complex that it may place a postural threat on an individual, or if the individual is not capable of performing the task due to physical impairment, attentional resources are drawn from the cognitive task to the postural task to maintain balance [3, 28]. This strategy would be a safety measure to prevent a fall and is commonly seen in older adults, people with impaired balance [26] or when the physical task is challenging [13, 29]. The results of this study may imply that stroke survivors prioritize the physical task over the cognitive task to maintain balance during turning.

The result that stroke survivors prioritized the physical task over the cognitive one was inconsistent with previous studies that showed a compromised turning-while-walking performance when dual-tasking [14–17]. A possible explanation for this discrepancy is the instruction given on task prioritization. In the studies by Manaf’s research team, subjects were instructed to prioritize the cognitive task over the physical task [14–16]. Conversely, subjects in our study were told that both tasks were equally important and the task priority was not specified. It has been proposed that directing the priority of tasks may affect an individual’s response to a dual-tasking challenge [17]. In the absence of a specific instruction about the priority of tasks, stroke survivors were able to naturally react to the dual-tasking challenge. In addition, the nature of the cognitive task may affect the results of dual-tasking. In daily life, disturbances to physical movement could be triggered internally by an individual or externally by the environment. The cognitive task used in previous studies [14–17], serial subtraction, involves an internal interfering factor [30] so that subjects initiate the cognitive challenge without being prompted. By contrast, the auditory Stroop test requires the subject to react to an external event. The results of this study imply that the nature of the cognitive task plays a role in the interaction between the cognitive task and the physical task. However, further studies are needed to investigate the effect of instruction on task prioritization and the nature of cognitive task on dual-tasking performance in stroke survivors.

The second hypothesis that the dual-tasking performance of the stroke survivors would be worse than that of the controls was supported by this study. According to the theory of competition for attentional resources when dual-tasking, the increased demand for resources to conduct the individual tasks has a detrimental effect on dual-tasking performance [12, 26, 27]. In this study, the lower level of single-tasking performance of both the cognitive task and physical task found in stroke survivors may therefore contribute to the decreased dual-tasking ability, at least in such task combination. However, the exact mechanism that explains the poorer dual-tasking performance revealed in stroke survivors requires further investigation.

Stroke survivors in this study turned with a longer duration and a higher number of steps than the controls. This difference can be attributed to various factors such as gait asymmetry and lower limb weakness, which is common in stroke survivors. In a 180-degree turn, the inner leg acts as the axis of the turn and accepts the entire body weight. The outer leg then swings for a longer time and travels a longer distance than in straight line walking [31]. However, if the ability of the inner leg to bear the body weight is diminished, the single leg stance time would then decrease. The time and distance for the outer leg to travel would therefore be reduced. As a result, a longer time and a greater number of steps should be made to complete the turn. This may explain the difference in turning performance between stroke survivors and controls found in this study. Furthermore, the mean turning duration for stroke survivors in this study was more than 3.18 ± 0.28 sec, suggesting that they exhibited some difficulty while turning [4].

The dual-task cost has been used to compare the change in performance between single-tasking and dual-tasking with the difference in single-tasking performance among the subjects being considered [25]. The insignificant between-group difference for the dual-task cost may indicate that when taking the variation in single-tasking performance into account, the demand for attentional resources when dual-tasking were similar between stroke survivors and controls.

### Limitations

One of the limitations of this study was that subjects with a stroke were relatively mobile; they were able to walk and turn unaided indoors. Besides, the mean MMSE score was 27.9 out of 30. Hence, the results of this study only apply to stroke survivors with relatively high global cognitive functioning and physical function. Further research including stroke survivors with lower cognitive and physical functions is needed to improve the generalization of the study results. Another limitation was the uneven distribution of gender between the two groups. In addition, the stroke survivors were significantly taller than the controls. The statistical analyses were conducted again by adding the gender and body height as covariates. The statistical results remained similar with the covariates added. It is therefore reasonable to assume that gender and height differences do not affect the findings of this study.

### Clinical relevancy

This study showed a poorer dual-tasking performance when turning-while-walking is the physical task in stroke survivors than in age-matched counterparts. The results suggest that such dual-tasking test may usefully be included in clinical practice, especially for those with turning difficulty. In addition, the number of steps for stroke survivors to complete the turn was relatively high, which may imply the presence of turning difficulties [4]. Given the risk of falling when turning [3] and its potentially severe consequences [1], this activity may be included in rehabilitation programs for stroke survivors.

### Conclusions

Results from this study suggest that stroke survivors compromise cognitive task performance to perform the turning-while-walking task when dual-tasking. The behavior can be explained by the theory of competition for attentional resources and the posture-first strategy. These results differ from those of previous studies. Variations in the instruction of task prioritization and the nature of the cognitive task may explain the discrepancy. However, further studies must be carried out to examine this hypothesis.

Additionally, stroke survivors had a longer reaction time and a lower accuracy for the cognitive task when both single-tasking and dual-tasking as compared with controls. They also turned with a longer duration and more steps and took a longer time to complete the turning-while-walking task under both tasking conditions. However, the dual-task cost of all the parameters was similar between the two groups. The degraded single-tasking ability after a stroke may contribute to the poorer dual-tasking performance, but further investigation is needed to find out the exact mechanism of such phenomenon.
